# Health and Well-Being of Adolescents in Different Family Structures in Germany and the Importance of Family Climate

**DOI:** 10.3390/ijerph17186470

**Published:** 2020-09-05

**Authors:** Max Herke, Anja Knöchelmann, Matthias Richter

**Affiliations:** Institute of Medical Sociology, Martin-Luther-University Halle-Wittenberg, 06112 Halle (Saale), Saxony-Anhalt, Germany; anja.knoechelmann@medizin.uni-halle.de (A.K.); m.richter@medizin.uni-halle.de (M.R.)

**Keywords:** subjective health, well-being, family structure, family climate, children, adolescents, National Educational Panel Study, Germany

## Abstract

The family is of exceptional and lifelong importance to the health of adolescents. Family structure has been linked to children’s and adolescents’ health and well-being; a nuclear family has been shown to be indicative of better health outcomes as compared with a single-parent family or a step-family. Family climate is rarely included in studies on children’s and adolescents’ health and well-being, albeit findings have indicated it is importance. Using data from *n* = 6838 students aged 12–13 years from the German National Educational Panel Study, this study shows that stronger familial cohesion and better a parent-child relationship are associated with better self-rated health, higher life satisfaction, more prosocial behavior, and less problematic conduct, and that these associations are stronger than those for family structure. Surveys on young people’s health are encouraged to include family climate above and beyond family structure alone.

## 1. Introduction

The family is of exceptional and lifelong importance to the health of adolescents, both physical and mental health, as well as health behaviors [1,2]. In childhood and adolescence, social determinants such as income, education, or occupational status are often derived from the family and influence health. Persons in advantaged socioeconomic positions are less likely to suffer from illness and other health impairments. But there are other determinants of children’s and adolescents’ health stemming from the family, such as family structure or the family climate, especially the latter being rarely examined and underdeveloped. The family structure is described by the family members present in a child’s or adolescent’s household, whereas the family climate refers to the interpersonal relationships within the family. Theoretical approaches on how these determinants stemming from the family are linked to adolescents’ health exist alongside and complement each other.

First, the family provides resources. Parents often introduce their individual socioeconomic resources into the household and provide their children and each other with life chances. These resources are often due to the socioeconomic background, material resources, occupational position, or the education of the parents. More resources are linked to better physical and mental health [3]. Adolescents in socially advantaged positions have more resources at their disposal, and therefore more health-related opportunities.

Second, the family provides social support and integration to compensate for external psychosocial burdens and provides an important context in which to learn, practice, and also assert health behaviors [4]. Social support includes emotional, instrumental, or informational support for each other, as well as social integration, all of which are factors of resilience and important family functions [5], which can act as a buffer to compensate external stressors (“buffering hypothesis”, [6]), for example, by lessening the impact of stressful events. Furthermore, close social relationships convey a sense of meaningfulness and a sense of belonging to a community, and thereby benefit health [7]. Adolescents receiving the necessary social support to compensate for stress are more likely to maintain good physical and mental health [8].

Third, the family creates social control, which is another dimension of social integration [9]. The link between social integration and health is established via health behaviors and it is suggested that family members have a deterrent effect on negative health behaviors, which in turn would affect health negatively [9,10]. Adolescents well integrated into their families experience stronger social control, which could prevent them from engaging in activities detrimental to their health.

Research on the relationship between family and health has a long history and repeatedly confirms that family structures and health are linked [11]. Nuclear families, single-parent families, and step-families are the most common family structures and refer to living with both (biological) parents (nuclear family), living with only one parent (single-parent families), or living with one parent and one non-biological or step-parent (step-families) in this study, omitting rarer family structures. Studies have shown that adolescents who were not living in nuclear families and, in particular, those living in single-parent families reported more health complaints, chronic diseases, and worse health-related quality of life, as well as had a worse assessment of health by their parents [12,13,14,15,16]. Children and adolescents that did not live in nuclear families have also reported lower life satisfaction or mental well-being, were more likely to report mental problems [17,18,19,20,21,22,23,24], and showed significantly more unfavorable internalizing or externalizing behaviors [12,17,25,26]. Findings on the connection between health and changes in family structures due to family transitions are less common. At the international level, a few studies with current and representative longitudinal data can be found and some are summarized in various reviews [27,28,29,30,31,32], however, predominantly from North American conditions. For Germany, research on this exists only in part and only on divorces and separations [33,34,35]. The findings were inconsistent and mostly indicate little to no short-term consequences for physical health, but numerous psychosocial problems that arose for adolescents.

The family climate is based on the family relationships, which also influence the mental and physical health of family members [36]. The family climate includes many different aspects and we refer to a simple model that distinguishes two main dimensions. On the one hand, there are the more relationship-related aspects such as cohesion, communication, or conflicts, and, on the other hand, there are the more family system-related aspects such as organization or control [37]. Few studies have accounted for associations of aspects of family climate with health, even though a good family climate has been shown to be associated with better health in children and adolescents. Familial cohesion and stability are resources that have also been positively associated with health for adolescents [8,13] and to improved adjustment after parental divorce or separation [35,38,39].

Socioeconomic background and its associations with adolescent health have been extensively researched [3]. It is important to note that socioeconomic resources are closely linked to family structure. In 2016, a total of 30.7% of all households of the member states of the European Union had adults with children. Couples with children represented 20.5%, single adults with children 4.3%, and other types of households where grandparents and parents lived together with children represented 5.8% of all households [40]. Single-parent families, especially, are more likely to have lower educational backgrounds and to live in relative income poverty. Therefore, researches that examine family structure and how it is linked to adolescents’ health usually control for socioeconomic background. However, social support and social control are rarely accounted for. 

In summary, the effects of family transitions on physical health are small, more pronounced in mental health outcomes, and are most strongly reflected in problematic and potentially unhealthy behavior. Although, for example, there is a strong selection effect involved, generally, due to the less favorable socioeconomic background of those families in which the parents are divorced or separated, there is also a connection between these transitions and changes in the health of adolescents, as well as numerous differential effects. The timing of family transitions is relevant, i.e., the age at which adolescents experience the transition [41,42,43]. There are also gender differences [41,43,44,45], i.e., which parent leaves the family [46] and the gender of the parents and of the child [25,47,48,49], as well as the relationship the child has with the parent who is not living in the household [25]. Children’s resilience can also play a role [27,50].

Reviewing this state of research, it becomes apparent, that there is a strong emphasis on family structure, whereas research on family climate’s impact on health is underdeveloped. Furthermore, family structure is a result of family transitions and also easier to measure but does not depict the complex processes in transitions, and therefore is only a proxy for the better understood impact of family transitions. Few cross-sectional, as well as longitudinal studies, point toward the possible importance of the family climate, beyond the simple configuration of individuals within a family unit. Therefore, this study investigates how family structure and family climate are linked to adolescents’ health.

This study uses data on adolescents aged 12 to 13 years from the “National Educational Panel Study” (NEPS; [51]). This study focuses on the family structure of the adolescents, and differentiates among nuclear families, single-parent families, and step-families, as well as considers two aspects of family climate, i.e., familial cohesion and parent-child relationship. We examine how these are linked to self-rated health [52], the “Strengths and Difficulties Questionnaire” [53] scales for prosocial behavior and problematic conduct, as well as overall life satisfaction. We aim to answer the following three questions:

1. Do adolescents who grow up in nuclear families, as compared with adolescents who grow up in single-parent families or step-families, show better health?
2. Do adolescents who report a better family climate show better health?
3. What is the relative contribution of family structure as compared with family climate to adolescents’ health?

## 2. Materials and Methods 

### 2.1. Data and Sample

The “National Educational Panel Study” (NEPS) is carried out by the Leibniz Institute for Educational Trajectories (LIfBi) at the University of Bamberg. It is designed to examine educational processes in Germany across the entire lifespan and is part of the “Framework Program for the Promotion of Empirical Educational Research” funded by the German Ministry of Education and Research and supported by federal states [51]. It is comprised of six separate cohorts starting in 2010. Students were originally sampled through a stratified multistage process. After stratification by school type, region, and other characteristics, schools were drawn and then full classes were sampled [54]. It provided valuable indicators, not only on subjective health, but also on sociodemographic and socioeconomic characteristics and included data obtained from the parents.

The analyses drew upon the data from students attending regular schools of the “Starting Cohort 3 ‘5th graders’” (SC3). The sampling process followed a multistep, stratified design. In the first step, schools were sampled from a comprehensive list of schools in Germany. Data on school type and region were used for stratification of this sample, to replace non-participating schools with draws of similar schools, as NEPS intends to provide a representative sample of German schools. In the second step, two or more classes from each participating school were sampled. All students within these classes were asked to participate, as well as selected teachers (class, math and German teachers), as well as students’ parents [55,56]. We used data from the third survey wave from the fall and winter of 2012, when most students were in the seventh grade (*n* = 6838 realized student interviews in 710 classes, nested in 277 schools). An additional sample of schools for students with special educational needs was excluded, due to limited comparability of the survey instruments used and possible systematic differences in students attending these schools to those students attending regular schools.

Students were interviewed in class using paper and pencil interviews (PAPI) and their parents were interviewed using computer-assisted telephone interviews (CATI). The survey documents used were previously submitted to and reviewed and approved by the respective Ministries of Education of the 16 federal states. During the survey, NEPS worked closely with the relevant data protection officers of the federal states for strict compliance with the statutory data protection regulations [51].

### 2.2. Indicators

Table 1 presents the sample description and an overview of the variables used in this study. In addition to the possible values or range of values, it provides relative frequencies or means and standard deviations for all indicators. It also includes the sample description differentiated by family structure. Table S1 in Supplementary Materials contains the sample correlations, providing pairwise Pearson correlation coefficients for all variables used in this study.

#### 2.2.1. Dependent Variables

Self-rated health is used as a general indicator for children’s physical health, as it has been established in other child health surveys and correlates well with other measures of health, for example, mortality [52]. Its five response categories are dichotomized for the analyses to indicate poor self-rated health (“very good/good” = 0 versus “average/poor/very poor” = 1).

Life satisfaction as a measure of subjective well-being is of crucial importance to young people’s psychological, educational, social, and physical functioning [55], and therefore is used as an indicator of students’ well-being. It was measured using the Cantril ladder [56], asking students “How satisfied are you with your life?” (response options, 0 = “completely dissatisfied” to 10 = “completely satisfied”) and was included as a metric measure in the analyses.

The Strengths and Difficulties Questionnaire (SDQ) provided indicators for children’s mental health [53]. Two subscales of the SDQ are included in NEPS, which cover children’s “prosocial behavior” and “problematic conduct” and each one is measured with five items. The scales are included as metric measures in the analyses and higher values indicate more prosocial behavior or more problematic conduct, respectively.

#### 2.2.2. Independent Variables

The family structure was constructed analogous to the “Health Behaviour in School-aged Children” [57]. On the basis of the reports by the children regarding which persons live in the domestic household (mother, father, step-parents, grandparents, siblings, and other relatives) we distinguished nuclear families, single-parent families, and step-families, which were included as a categorical measure in the analyses, considering nuclear families as the reference category.

Family climate, in this study, used two indicators, which were included to account for aspects of social integration and control, as well as social support. The analyses included a scale for familial cohesion, which was constructed from five items (Cronbach’s α = 0.80) and a scale for parent-child relationship, which was constructed from three items (Cronbach’s α = 0.78). Family cohesion was measured asking “To what extent do the following apply to your family?” with regard to items such as “The members of our family are emotionally close to each other” or “In our family there is a strong sense of solidarity”. The parent-child relationship was measured asking “To what extent do the following statements apply to you and your parents?” for items such as “I can talk easily to my parents about what worries me” or “I feel that my parents take me seriously”. For both scales, higher values indicated stronger familial cohesion or a better parent-child relationship, respectively [37].

#### 2.2.3. Control Variables

We controlled for children’s sex, age, migration background, and school type. Children born outside of Germany or having at least one parent born outside of Germany were considered as having a migration background. The German school system is hierarchically organized into co-existing tracks during secondary education; most commonly distinguished are the highest track “Gymnasium”, the intermediate track “Realschule”, the lowest track “Hauptschule”, and a comprehensive track “Gesamtschule”, combining aspects of all. On the basis of achievement, students are given a school track recommendation after primary school; only the high track schools and corresponding tracks within the comprehensive track schools cover higher secondary education and the attainment of a university entrance qualification. The attended school type was introduced as a categorical variable, i.e., low track (“Hauptschule”), medium track (“Realschule”), and high track schools (“Gymnasium”), as well as mixed track schools (“Gesamtschule”) combining aspects of all tracks. Children attending the high track schools were considered to be the reference group. Furthermore, we controlled for parental education due to the likelihood of a selection process at work, as couples with lower educational status were more likely to get divorced [58,59]. We used the highest educational level of any parent or step parent living with the child, and then used the CASMIN-classification (Comparative Analysis of Social Mobility in Industrual Nations; Brauns, [60]) to differentiate a high (lower and higher tertiary education), medium (intermediate to general maturity certificate with or without vocational qualification), and low (inadequately completed general education, general elemental education, or basic vocational qualification) educational level.

### 2.3. Statistical Analysis

We used logistic regression models to examine poor self-rated health and linear regression models to examine the SDQ indicators for prosocial behavior and problematic conduct, and life satisfaction. As independent variables, we included family structure, familial cohesion, and parent-child relationship. As controls, we included age, gender, migration background, school type, and parental educational level. All metric measures were z-standardized before regression analyses.

In total, the sample contained 6838 realized student interviews. However, there was significant item nonresponse present, and an analysis of only complete cases would have led to a significant reduction of the effective sample size (53.7% of the cases had at least some missing data, even though only 11.0% of all data was missing), which was not deemed feasible. Furthermore, item nonresponse was strongly associated with some of the independent variables, i.e., missing information is more likely for students in single-parent families or step-families (χ^2^ = 44.474, df = 2, *p* < 0.001) and for students reporting weaker familial cohesion (F = 8.673, df = 1, *p* < 0.004), or worse parent-child relationship (F = 12.516, df = 1, *p* < 0.001). Therefore, we assumed the mechanism producing missing data was not missing completely at random (MCAR) [61], which would be necessary to produce unbiased estimates when analyzing only complete cases.

We assumed the mechanism leading to missing values to be missing at random (MAR), and therefore integrated multiple imputations into the analyses to minimize bias stemming from missing data. This method was very well suited to this task, regarding the sample size, the number of variables included in the imputation model, and the analyses to be conducted [61,62,63,64]. The imputation model contained raw data for all variables used in the analysis, before items were combined into scales or dichotomized. The imputation model used logistic methods for dichotomous and categorical variables and predictive mean matching (PMM) to impute ordinal or metric variables [65]. We imputed 100 datasets, balancing appropriate statistical power [66] with computational expenditure of time.

For dichotomous outcomes, we reported odds ratios, 95% confidence intervals, and *p*-values from logistic regressions, and for continuous outcomes we reported β (standardized beta coefficients), standard errors, and *p*-values from linear regressions. We used R for all analyses [67] and the package “mice” (“multiple imputation by chained equations”, [68]) for imputation and combination of results.

## 3. Results

Self-rated health shows no associations with the family structure. However, children with a stronger familial cohesion or a better parent-child relationship are less likely to report poor self-rated health (familial cohesion, OR = 0.79, CI 95% = 0.72–0.88, *p* < 0.001; parent-child relationship, OR = 0.74, CI 95% = 0.68–0.82, *p* < 0.001). Furthermore, poor self-rated health is more likely in girls and students attending low track schools (see Table 2). The average marginal effects for this logistic regression are reported in Table S2 in Supplementary Material.

Children show lower life satisfaction when they are living in a single-parent family (β = −0.09, SE = 0.035, *p* < 0.015) or in a step-family (β = −0.14, SE = 0.038, *p* < 0.001). Children engaged in better parent-child relationships and stronger familial cohesion, report a higher satisfaction with life (familial cohesion, β = 0.22, SE = 0.016, *p* < 0.001; parent-child relationship, β = 0.29, SE = 0.016, *p* < 0.001). In addition, life satisfaction is lower for girls and students attending the medium track but is higher for children with a migrant background. See Table 2.

Prosocial behavior in adolescents shows no differences by family structure, but it is more pronounced in children with stronger familial cohesion (β = 0.16, SE = 0.021, *p* < 0.001) and better parent-child relationships (β = 0.13, SE = 0.021, *p* < 0.001). Prosocial behavior is also more pronounced in girls, but less pronounced in students attending medium or mixed track schools. Children show more problematic conduct when living in single-parent families (β = 0.10, SE = 0.049, *p* < 0.049) or step-families (β = 0.13, SE = 0.054, *p* < 0.017), but less problematic conduct with stronger familial cohesion (β = −0.11, SE = 0.022, *p* < 0.001) and better parent-child relationships (β = −0.05, SE = 0.022, *p* < 0.020). Girls show less problematic conduct, and students attending any other school type than high track schools show more problematic conduct (see Table 3).

## 4. Discussion

First, adolescents growing up in nuclear families do not show better self-rated health or prosocial conduct than others. However, adolescents growing up in a single-parent family or a step-family show slightly lower life satisfaction and more problematic conduct. Second, a good family climate is associated with better health and well-being. Adolescents reporting tighter familial cohesion or a better parent-child relationship also show better self-rated health, higher life satisfaction, more prosocial behavior, and less problematic conduct. Third, the actual family structure that children live in is less important as compared with the family climate.

On the one hand, the findings regarding associations of family climate with health and well-being are well in line with some of the few studies that incorporated family climate [8,13]. Other studies have also found negligible or no associations between family climate and health and well-being [69], or even only controlled for family climate [8,12]. Most of these studies were also methodologically limited, as they employed single item to operationalize family climate, instead of scales modeled towards a well-developed conceptual framework. On the other hand, the findings on the association of family structure with health and well-being do not clearly contradict existing findings, but also do not put as much emphasis on family structure.

A final and definitive allocation as to which of the theoretical approaches is best suited to describe how family is linked to children’s health is not possible within the scope of this paper and the operationalizations available. However, the different indicators lend themselves more to some of the theoretical approaches than others. Family structure is more closely linked to resources. Step-parents and, in particular, single parents are at a higher risk of living in income poverty [70,71], and parents with more unfavorable social background characteristics are also more likely to get divorced or separated [58,59]. A theoretical model by Amato [28] explicitly referred to the case of divorce or separation of adult partners and postulated that there were immediate social and economic changes as well as spatial mobility as a result of the divorce or separation, because conflicts, separation of property, moving out, and more, arose directly from that change. These stressors are often examined as social determinants of health and are embedded in models explaining the mechanisms and causes of health inequalities in childhood and adolescence. In the theoretical model, the stressors take on the function of mediators, which convey the influence of changes in the family structure on individual characteristics such as physical and mental health. However, as summarized in the introduction, long-term effects of these family transitions, which are reflected in the family structure, ultimately show only small associations with health and well-being [27,28,29,30,31,32]. Furthermore, family climate plays an important role in family processes and can act as moderators for the impact of stressors on health and well-being [35,38,39]. However, familial cohesion and parent-child relationship show only minimal correlations with family structure (see Table S1) and are more closely linked to social support and social control. Family climate is related to familial systems, mostly shaped by the experiences within, and thus serves as a global indicator of the quality of all intrafamilial interactions [37], which contain social support and control. Considering the minimal correlations between family structure and family climate, both should be considered to be determinants of health.

This study is based on high quality data, as NEPS provides a current, representative, and large sample. In this study, we were able to address important problems of selectivity, especially considering the associations of family structure with socioeconomic status. Sensitivity analyses were carried out to investigate the validity of the analytical model. A sensitivity analysis repeats the primary analysis, but substitutes alternatives in the modeling, for example, in the operationalization or inclusion of certain variables, to test the robustness of findings and the consequences of decisions on the analytical strategy. First, we tested models that only included family structure, and then consecutively added family climate indicators. The impact of family structure was significantly greater only for life satisfaction, in the model excluding family climate indicators. The inclusion of family climate indicators reduced the impact of family structure. Furthermore, the two indicators of family climate showed some signs of multicollinearity, but only slightly to moderately reduced coefficients for these two independent variables. Second, we tested models using only the control variables. This yielded results consistent with existing findings. Possible biases due to missing data were addressed by using multiple imputations. Unfortunately, this study shares weaknesses with other studies in terms of the operationalization of family structure. The family structure is derived from reports of the children on their main household, which might not accurately represent the actual family composition. Due to the circumstance that many of the health outcomes or indicators of family structure and climate were only obtained at singular or otherwise unsuitable time points, longitudinal analyses were not possible, and this study relied on cross-sectional analyses, even though NEPS provided longitudinal data. Therefore, when interpreting these associations, we could not clearly differentiate between selection and causality. Further data releases including additional panel waves could mitigate this weakness of the NEPS data.

## 5. Conclusions

A good family climate is strongly associated with good health and well-being in children as compared with the actual family structure children live in which is of lesser importance, when controlling for core determinants of socioeconomic status, for example, school type (which is strongly linked to socioeconomic status in Germany) or parental education. However, this is not yet represented in the majority of studies examining the impact of the family on children’s and adolescents’ health and well-being.

Parental divorce or separation is a long-term developmental process, often taking years. In the underlying explanatory model [28], separation and other family transitions themselves had minimal direct effects on the affected children’s or adolescents’ well-being, but the accompanying stressors often increased the likelihood of psychological, behavioral, and health problems. The static family structure measured at any given time in youth health surveys is a result of such family transitions. Family structure, and more specifically, living in a non-nuclear family, often is considered to be a risk factor, but it is only considered to be a distant proxy to the actual stressors associated with family transitions, for example, long-lasting conflicts, a decline in material resources due to the splitting of households, moving, or associated loss of social resources. Furthermore, many factors moderate children’s and adolescents’ reactions and their adjustment to parental separation and other family transitions. How these transitions impact the well-being of children is moderated by factors such as individual and interpersonal resources, specifics in the structural changes, timing, gender, siblings, resilience, and more [25,27,41,42,43,44,45,48,49,50,72]. Accounting only for socioeconomic status, in addition to family structure, is insufficient to assess what is ultimately the consequence of stressors that come along with family transitions. Additional factors should be considered, and family climate could provide good measures for some of the social resources available to children and adolescents.

Although the inclusion of indicators of family climate is becoming more widespread, the scope of most studies on child and adolescent health and health inequalities that consider family are still restricted to family structure and possibly indicators on familial socioeconomic status at best. To more consistently expand this scope, we suggest including family climate and using either existing indicators or developing new indicators that could be more suitable. 

## Figures and Tables

**Table 1:** Sample description (National Educational Panel Study, Starting Cohort 3, Wave 3, 2012, *n* = 5769).

Variable	Values	Students (*n* = 5769; 78.1%)	Students by Family Structure			Percent Missing
			Nuclear Family (*n* = 4328; 11.7%)	Single-Parent Family (*n* = 788; 10.2%)	Step-Family (*n* = 653)	
Dependent						
Poor self-rated health (%)	0, 1	15.4%	14.6%	16.2%	19.6%	2.7%
SDQ prosocial behavior (x, σ^2^)	0–10	7.4 (1.9)	7.4 (1.9)	7.2 (1.9)	7.2 (1.9)	27.3%
SDQ problematic conduct (x, σ^2^)	0–10	2.3 (1.8)	2.2 (1.8)	2.6 (2.3)	2.7 (2.0)	28.5%
Life satisfaction (x, σ^2^)	0–10	7.5 (2.1)	7.7 (2.0)	7.1 (2.3)	7.0 (2.4)	1.8%
Independent						
Familial cohesion (x, σ^2^)	5–25	18.6 (3.8)	19.0 (3.6)	17.4 (4.1)	17.4 (4.0)	5.2%
Parent-child-relationship (x, σ^2^)	3–15	12.3 (2.6)	12.5 (2.5)	11.9 (2.8)	11.8 (2.7)	3.1%
Control						
Age (x, σ^2^)	9–16	12.5 (0.6)	12.4 (0.6)	12.6 (0.7)	12.6 (0.6)	<0.1%
Sex (% female)	0, 1	49.6%	50.1%	49.6%	47.0%	<0.1%
Migration background (%)	0, 1	17.2%	17.1%	20.4%	13.2%	5.5%
School type						9.4%
High track (%)	0, 1	48.2%	52.5%	36.0%	34.5%	-
Medium track (%)	0, 1	26.1%	24.7%	29.1%	31.5%	-
Low track (%)	0, 1	6.5%	5.3%	10.8%	9.0%	-
Mixed track (%)	0, 1	9.8%	8.1%	14.2%	15.8%	-
Parental education	-	-	-	-	-	37.8%
High	0, 1	22.5%	25.7%	13.1%	12.6%	-
Medium	0, 1	34.5%	34.8%	31.7%	36.4%	-
Low	0, 1	5.2%	4.3%	8.4%	6.9%	-

Notes: x = mean; σ^2^ = standard deviation.

**Table 2:** Results for poor self-rated health (logistic regression) and life satisfaction (linear regression).

Variable	Poor Self-Rated Health			Life Satisfaction		
	OR	CI 95%	*p*	β	SE	*p*
Intercept	0.13 ***	0.11–0.16	<0.001	0.08 **	0.025	<0.003
Independent						
Family structure (ref. = nuclear family)						
Single-parent family	0.88	0.70–1.10	0.256	−0.09 *	0.035	0.015
Step-family	1.11	0.89–1.39	0.362	−0.14 ***	0.038	<0.001
Familial cohesion	0.79 ***	0.72–0.88	<0.001	0.22 ***	0.016	<0.001
Parent-child-relationship	0.74 ***	0.68–0.82	<0.001	0.29 ***	0.016	<0.001
Control						
Age	1.05	0.97–1.13	0.219	−0.01	0.012	0.483
Gender (ref. = male)	1.27 **	1.09–1.47	0.003	−0.11 ***	0.023	<0.001
Migration background	0.85	0.69–1.04	0.113	0.10 **	0.031	0.002
School type (ref. = high track)						
Medium track	1.11	0.92–1.33	0.265	−0.07 *	0.029	0.022
Low track	1.38 *	1.03–1.87	0.035	−0.04	0.050	0.471
Mixed track	1.23	0.96–1.57	0.106	−0.07	0.040	0.103
Parental education (ref. = high)						
Medium	1.11	0.93–1.33	0.232	0.02	0.027	0.420
Low	1.05	0.78–1.40	0.743	0.01	0.046	0.790

Notes: Life satisfaction, familial cohesion, parent-child relationship, and age are z-standardized; * *p* < 0.05; ** *p* < 0.01; *** *p* < 0.001; OR, odds ratio; CI 95%, 95% confidence interval; *p*, *p* value; ref., reference group.

**Table 3:** Results for the Strengths and Difficulties Questionnaire (SDQ) scales, prosocial behavior and problematic conduct (linear regressions).

Variable	SDQ: Prosocial Behavior			SDQ: Problematic Conduct		
	β	SE	*p*	β	SE	*p*
Intercept	−0.24 ***	0.031	<0.001	−0.08 *	0.033	0.014
Independent						
Family structure (ref. = nuclear f.)						
Single-parent family	0.00	0.047	0.993	0.10 *	0.049	0.049
Step-family	0.04	0.051	0.435	0.13 *	0.054	0.017
Familial cohesion	0.16 ***	0.021	<0.001	−0.11 ***	0.022	<0.001
Parent-child relationship	0.13 ***	0.021	<0.001	–0.05 *	0.022	0.020
Control						
Age	0.02	0.016	0.361	− 0.03	0.017	0.124
Gender (ref. = male)	0.54 ***	0.030	<0.001	−0.16 ***	0.032	<0.001
Migration background	0.05	0.040	0.230	0.08	0.043	0.058
School type (ref. = high track)						
Medium track	−0.09 *	0.038	0.018	0.24 ***	0.040	<0.001
Low track	−0.11	0.063	0.091	0.35 ***	0,066	<0.001
Mixed track	−0.17 ***	0.050	<0.001	0.28 ***	0.053	<0.001
Parental education (ref. = high)						
Medium	0.01	0.060	0.859	0.02	0.036	0.306
Low	0.07	0.034	0.240	−0.07	0.064	0.564

Notes: Prosocial behavior, problematic conduct, familial cohesion, parent-child relationship, and age are z-standardized; * *p* < 0.05; *** *p* < 0.001; β, standardized beta coefficient; SE, standard error; *p*, *p* value; ref., reference group.

## Supplementary Materials

The following are available online at https://www.mdpi.com/1660-4601/17/18/6470/s1, Table S1: Sample correlations (National Educational Panel Study, Starting Cohort 3, Wave 3, 2012, *n* = 5769), Table S2: Logistic regression reporting marginal effects for poor self-rated health.
