# Supplementary material for: Health and Well-Being of Adolescents in Different Family Structures in Germany and the Importance of Family Climate
Source: Int J Environ Res Public Health. 2020 Sep 5;17(18):6470. doi: 10.3390/ijerph17186470 (PMC7559242; doi:10.3390/ijerph17186470)
Supplement: Supplementary file 1 [file ijerph-17-06470-s001.pdf]

Table S1. Sample correlations (National Educational Panel Study, Starting Cohort 3, Wave 3, 2012, *n* = 5,769).

	**(1)**	**(2)**	**(3)**	**(4)**	**(5)**	**(6)**	**(7)**	**(8)**	**(9)**	**(10)**	**(11)**	**(12)**	**(13)**	**(14)**	**(15)**	**(16)**	**(17)**	**(18)**	**(19)**
Dependent																			
(1) Poor self-rated health																			
(2) Life satisfaction	**−0.25**																		
(3) SDQ: Prosocial behavior	**−0.04**	**0.15**																	
(4) SDQ: Problematic conduct	**0.14**	**−0.23**	**−0.13**																
Independent																			
Family structure																			
(5) Nuclear family	**−0.04**	**0.12**	**0.05**	**−0.10**															
(6) Single-parent family	0.01	**−0.07**	**−0.04**	**0.06**	**−0.69**														
(7) Step-family	**0.04**	**−0.09**	−0.03	**0.06**	**−0.62**	**−0.14**													
(8) Familial cohesion	**−0.03**	0.00	0.02	−0.01	0.00	0.00	0.00												
(9) Parent-child-relationship	−0.02	0.01	0.02	0.01	0.01	0.01	−0.02	**0.68**											
Control																			
(10) Age	**0.03**	**−0.03**	−0.03	0.02	**−0.10**	**0.08**	**0.05**	0.01	0.01										
(11) Gender (ref. = Male)	**0.04**	**−0.05**	**0.27**	**−0.08**	0.01	0.00	−0.02	−0.01	−0.02	**−0.08**									
(12) Migration background	0.00	0.01	0.02	0.02	0.01	**0.03**	**−0.04**	−0.03	**−0.03**	**0.08**	0.01								
School type																			
(13) High track	**−0.05**	**0.08**	**0.07**	**−0.15**	**0.16**	**−0.11**	**−0.11**	**0.03**	0.02	**−0.21**	**0.04**	**−0.08**							
(14) Medium track	0.02	**−0.05**	−0.03	**0.07**	**−0.06**	**0.03**	**0.05**	−0.02	0.00	**0.10**	−0.02	0.02	**−0.68**						
(15) Low track	**0.03**	**−0.03**	**−0.04**	**0.09**	**−0.09**	**0.07**	**0.04**	0.01	0.02	**0.14**	**−0.04**	**0.08**	**−0.30**	**−0.18**					
(16) Mixed track	**0.03**	−0.03	**−0.04**	**0.07**	**−0.10**	**0.06**	**0.07**	**−0.03**	**−0.04**	**0.07**	0.01	**0.05**	**−0.37**	**−0.22**	**−0.10**				
Parental education																			
(17) High	**−0.04**	**0.05**	0.01	**−0.06**	**0.14**	**−0.09**	**−0.10**	−0.01	0.01	**−0.14**	0.01	**−0.12**	**0.33**	**−0.25**	**−0.14**	**−0.07**			
(18) Medium	0.03	−0.03	−0.02	0.04	**−0.07**	0.03	**0.07**	0.00	−0.01	**0.06**	0.00	**0.07**	**−0.18**	**0.15**	0.03	**0.04**	**−0.84**		
(19) Low	0.01	−0.03	0.01	**0.04**	**−0.11**	**0.10**	**0.05**	0.01	0.00	**0.14**	−0.01	**0.08**	**−0.26**	**0.16**	**0.19**	**0.04**	**−0.23**	**−0.34**	

Notes: Pearson correlation coefficients; pairwise correlations; nominal and ordinal indicators transformed into dummy-variables; **bold** = coefficients with $p < 0.05$; ref. = reference group.

Table S2. Logistic regression reporting marginal effects for poor self-rated health.

	Poor self-rated health		
	ME	SE	p
Intercept			
Independent			
Family structure (ref. = Nuclear f.)			
Single-parent family	−0.015	0.013	0.238
Step-family	0.013	0.015	0.375
Familial cohesion	−0.029 ***	0.006	<0.001
Parent-child-relationship	−0.037 ***	0.005	<0.001
Control			
Age	0.006	0.009	0.198
Gender (ref. = Male)	0.028 **	0.006	0.003
Migration background	−0.007	0.022	0.113
School type (ref. = High track)	0.036		
Medium track	0.013	0.012	0.293
Low track	0.036	0.022	0.121
Mixed track	0.032	0.017	0.080
Parental education (ref. = High)			
Medium	0.012	0.011	0.342
Low	0.007	0.019	0.543

** $p < 0.01$; *** $p < 0.001$.
